# Metabolomic prof iles of Ribes nigrum L. and Lonicera caerulea L. from the collection of the N.I. Vavilov Institute in the setting of Northwest Russia

**DOI:** 10.18699/VJGB-22-77

**Published:** 2022-11

**Authors:** T.V. Shelenga, V.S. Popov, A.V. Konarev, N.G. Tikhonova, O.A. Tikhonova, Yu.A. Kerv, A.E. Smolenskaya, L.L. Malyshev

**Affiliations:** Federal Research Center the N.I. Vavilov All-Russian Institute of Plant Genetic Resources (VIR), St. Petersburg, Russia; Federal Research Center the N.I. Vavilov All-Russian Institute of Plant Genetic Resources (VIR), St. Petersburg, Russia; Federal Research Center the N.I. Vavilov All-Russian Institute of Plant Genetic Resources (VIR), St. Petersburg, Russia; Federal Research Center the N.I. Vavilov All-Russian Institute of Plant Genetic Resources (VIR), St. Petersburg, Russia; Federal Research Center the N.I. Vavilov All-Russian Institute of Plant Genetic Resources (VIR), St. Petersburg, Russia; Federal Research Center the N.I. Vavilov All-Russian Institute of Plant Genetic Resources (VIR), St. Petersburg, Russia; Federal Research Center the N.I. Vavilov All-Russian Institute of Plant Genetic Resources (VIR), St. Petersburg, Russia; Federal Research Center the N.I. Vavilov All-Russian Institute of Plant Genetic Resources (VIR), St. Petersburg, Russia

**Keywords:** Ribes nigrum L., Lonicera caerulea L., VIR collection, nonspecif ic metabolomic prof iling, gas-liquid chromatography, mass spectrometry, fruit crops, biologically active substances, Ribes nigrum L., Lonicera caerulea L., коллекция ВИР им. Н.И. Вавилова, неспецифическое метаболомное профилирование, газожидкостная хроматография, масс-спектрометрия, плодовые культуры, биологически активные вещества

## Abstract

Recently, the trend of using fruit and berry crops as ingredients for functional and dietary nutrition, the development and implementation of f lavors, pigments, new medicines and dietary supplements has been actualized. Because the direction of use depends on the biochemical properties of fruits, which are determined not only by species and varietal characteristics, but also by reproduction conditions, the study of the biochemical composition of fruits grown in various regions of the world continues to be relevant. In this regard, the collection of N.I. Vavilov Institute (VIR), which has a wide diversity of fruit and berry crops, is of great interest for study. Ribes nigrum fruits have a balanced set of sugars, organic acids, essential oils, microelements, a high content of vitamins, anthocyanins, pectins. Lonicera caerulea fruits are characterized by high values of phenolic substances: biof lavonoids, hydroxycinnamic acids, f lavonols, polyphenols, anthocyanins, as well as vitamins, carotenoids, iridoid glycosides and other natural antioxidants. The investigation of L. caerulea and R. nigrum fruit’s accessions from the VIR collection using gas-liquid chromatography with mass spectrometry allows us to obtain new information about the biochemical characteristics of fruits, to identify L. caerulea and R. nigrum varieties with optimal economically valuable characteristics, to determine the specif icity of L. caerulea and R. nigrum metabolomic spectra in the setting of Northwest Russia. As a result of the analysis, typical compounds of the metabolomic prof ile of each culture were identif ied. Organic acids, phenol-containing compounds and polyols prevailed in L. caerulea, while mono- and oligosaccharides, in R. nigrum. The qualitative composition of the black currant varieties ‘Malen’kii Printz’, ‘Dobriyi Dzhinn’, ‘Tisel’, ‘Orlovskii Val’s’, and blue honeysuckle ‘S 322-4’, ‘Malvina’, ‘Leningradsky Velikan’ was optimal for food consumption; the varieties of blue honeysuckle ‘Bazhovskaya’ and black currant ‘Aleander’ had a good representation of biologically active compounds, which makes samples attractive as raw materials for the production of biologically active additives, including with the use of microorganisms’ cultures.

## Abstract (ru)

В последнее время актуализируются тенденция использования плодово-ягодных культур как ингредиентов для функционального и диетического питания, разработка и внедрение ароматизаторов, пигментов, новых лекарственных препаратов и биологически активных добавок. Направление применения зависит от биохимических характеристик плодов, которые обусловлены не только видовыми и сортовыми особенностями, но и условиями репродукции, поэтому исследование биохимического состава плодовых, выращенных в различных регионах мира, продолжает быть актуальным. В этой связи коллекция ВИР им. Н.И. Вавилова, обладающая широким разнообразием плодово-ягодных культур, представляет значительный интерес для изучения. Плоды Ribes nigrum отличаются сбалансированным набором сахаров, органических кислот, эфирных масел, микроэлементов, высоким содержанием витаминов, антоцианов, пектинов. Для плодов Lonicera caerulea характерны высокие значения фенолсодержащих веществ: биофлавоноидов, оксикоричных кислот, флавонолов, полифенолов, антоцианов, а также витаминов, каротиноидов, иридоидных гликозидов и других природных антиоксидантов. Исследование L. caerulea и R. nigrum из коллекции ВИР с применением газожидкостной хроматографии, сопряженной с масс-спектрометрией, позволяет получить новые сведения о биохимических характеристиках плодов, выделить сорта L. caerulea и R. nigrum с оптимальными хозяйственно ценными признаками, выявить специфику метаболомных спектров L. caerulea и R. nigrum в условиях Северо-Запада Российской Федерации. В результате анализа идентифицированы соединения, характерные для метаболомного профиля каждой из культур. У L. caerulea превалировали органические кислоты, фенолсодержащие соединения, полиолы, у R. nigrum – моно- и олигосахара. Качественный состав сортов черной смородины «Маленький Принц», «Добрый Джинн», «Tisel», «Орловский Вальс» и жимолости синей «С 322-4», «Мальвина», «Ленинградский Великан» оказался оптимальным для пищевого употребления. Сорта жимолости синей «Бажовская» и черной смородины «Алеандр» с хорошей представленностью биологически активных соединений привлекательны в качестве сырья для производства биологически активных добавок, в том числе с использованием культур микроорганизмов

## Introduction

Fruit crops represent a rich source of bioactive substan ces (BAS) with a broad range of properties beneficial for hu mans (Kylli, 2011). Recently, the trend of using fruit and berry crops as ingredients of functional and dietary foods, as well as for the development and introduction of flavors, pigments, new drugs and BAS has been gaining relevance (Konarev, Khoreva, 2000; Dudnik et al., 2018; Thole et al., 2019). The application depends on the biochemical characteristics of fruits, which are determined not only by characteristics of a species or variety, but also by the regeneration conditions, therefore, the study of the biochemical composition of fruits grown in different regions of the world maintains its relevance (Sochor et al., 2014; Gołba et al., 2020). In this regard, the collection of the N.I. Vavilov Institute of Plant Genetic Resources (VIR) that contains a wide range of fruit and berry crops is of interest for research.

Ribes nigrum is one of the most popular berry crops (Vit kovsky, 2003; Pikunova et al., 2011). To date, more than 1200 blackcurrant varieties have been bred and are cultivated (Knyazev, Ogoltsova, 2004). Lonicera caerulea has attracted attention relatively recently; its breeding has been actively developed since the 1940s–1950s. A great contribution to the promotion of blue honeysuckle was made by Prof. M.N. Ple khanova (VIR), the author of 24 varieties of L. caerulea (Plekhanova, 1992, 2000, 2007; Plekhanova, Streltsyna, 1998). The fruits of R. nigrum have a balanced set of sugars and organic acids, as well as a high content of vitamin C and dietary fiber (Dudnik et al., 2018; Thole et al., 2019; Tian et al., 2019). Honeysuckle is characterized by a high content of phenol-containing compounds (PCCs): bioflavonoids, hy droxycinnamic acids, flavonols, polyphenols, anthocyanins and other natural antioxidants. Also, the presence of iridoids is noted in its fruits (Senica et al., 2018; Gołba et al., 2020).

The purpose of the present study was to use gas-liquid chromatography coupled with mass spectrometry to obtain new information about the biochemical composition of fruits of R. nigrum and L. caerulea and to reveal the specifics of metabolomic profiles of fruits grown in conditions of the Le ningrad Province, to identify varieties with optimal eco nomically important characteristics, determine the prospects for the possible use of the selected accessions as raw material for expanding the range of products for functional and thera peutic nutrition, for the production of bioactive additives, as well as for breeding aimed at creating varieties that combine nutritional qualities with resistance to environmental stress factors.

## Materials and methods

The study was carried out on fruits of 20 R. nigrum and 10 L. caerulea accessions from the VIR collection grown in 2014 at the “Pushkin and Pavlovsk Laboratories of VIR” Research and Production Base located 30 km south of St. Pe tersburg. The blackcurrant varieties of Russian and foreign origin taken into the study included ‘Azhurnaya’, ‘Mura vushka’, ‘Orlovskii Val’s’, ‘Orlovskaya Serenada’, ‘Malen’kii Printz’, ‘Charovnitsa’, ‘Syuita Kievskaya’, ‘Cherechneva’, ‘Krasa L’vova’, ‘Ukrainka’, ‘Aleander’, ‘Pamyati Potapenko’, ‘Zhuravushka’, ‘Mila’, ‘Dobriyi Dzhinn’, ‘Slavyanka’, ‘Bi- ryu sinka’, ‘Volshebnitsa’, ‘Margo’, and ‘Tisel’, and those of blue honeysuckle included ‘Avacha’, ‘Start’, ‘Leningradsky Velikan’, ‘S 322-4’, ‘Malvina’, ‘Morena’, ‘Bazhovskaya’, ‘Su venir’, ‘Solovey’, and ‘838-12’. The material was grown according to the technique of E.N. Sedov and T.P. Ogoltso- va (1999). Meteorological conditions during the study were assessed as favorable for the vegetation of plants.

Each accession was represented by an average 50 g sample of fruits collected from three bushes at the stage of technical ripeness. The fruits were crushed in a Waring 800S laboratory blender (USA) in 100 mL of methanol (for HPLC, Vecton), centrifuged, and the supernatant was evaporated to dryness. The dry residue was silylated in 20 μL of bis(trimethylsilyl) trifluoroacetamide on Digi-Block (USA) for 15 min at 100 °C. The analysis was carried out in three analytical replications using an Agilent 6850A chromatograph coupled with an Agilent 5975 mass selective detector (USA) according to a protocol by Perchuk et al. (2020).

The obtained results were processed in the UniChrom and AMDIS programs using the NIST 2010 mass spectra libraries and in-house libraries of the Science Park of the St. Petersburg University and the V.L. Komarov Botanical Institute of the Russian Academy of Sciences (Puzanskiy et al., 2018; Shtark et al., 2019). The concentration was calculated in accordance with the recommendations by Worley and Powers (2013). The analytical data are presented in ppm (mg/kg) (Perchuk et al., 2020). The data were statistically processed in Statistica 7 and Excel 7.0 for Windows using factor analysis by the method of principal components and one-way analysis of variance.

## Results

The analysis of metabolomic profiles (MPs) of blackcurrant and honeysuckle has shown the presence of over 500 substances; less than 100 of them were precisely identified, and their indicators are presented in the article. In total, blackcurrant MPs were found to contain 88 and those of blue honeysuckle 75 components which belong to organic acids (39 and 29, respectively), free amino acids (2 and 3), polyols (6 and 7), free fatty acids (6 and 4), mono- and oligosaccharides (10 and 10; 4 and 5), sugar derivatives (7 and 4), and phenolcontaining compounds (14 and 11, respectively). In addition to the above substances, honeysuckle MPs contain choline and a purine derivative (1,2,3,6-tetrahydropurine-2,6-dione) (Suppl. Material 1)^1^.

Supplementary Materials are available in the online version of the paper: http://vavilov.elpub.ru/jour/manager/files/Suppl_Shelenga_27_7.pdf


The content of organic acids (ppm) in the studied honeysuckle fruit samples varied among varieties in the range from 78 383.85 (S 322-4) to 29 311.7 (Leningradsky Velikan), that of free amino acids from 705.2 (Malvina) to 32.4 (S 322-4), of polyols from 68 035.7 (Bazhovskaya) to 36 966.9 (Avacha), of pentoses from 8454.2 (S 322-4) to 2960.3 (Morena), of hexoses from 357 246.3 (S 322-4) to 171 672.8 (Avacha), of oligosaccharides from 63 824.1 (Leningradsky Velikan) to 7053.9 (Solovey), of glycosides from 3111.4 (Bazhovskaya) to 449.5 (Start), of free fatty acids from 588.4 (S 322-4) to 130.7 (838-12), and of PCCs from 29 353.3 (Bazhovskaya) to 11 001.2 (Start).

**Fig. 1.**
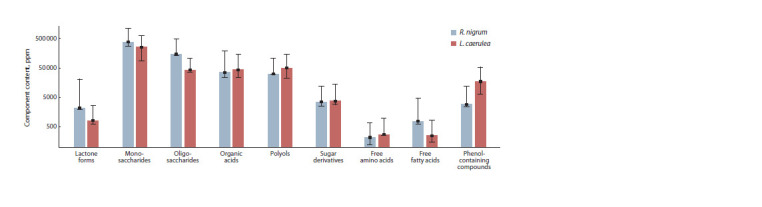
Main groups of compounds identified in the metabolomic profiles of fruit samples of R. nigrum and L. caerulea from the VIR collection.

In blackcurrant fruits, the range of variability was wider (ppm) for the following groups of compounds: from 110 551.4 (Aleander) to 13 743.7 (Orlovskii Val’s) for organic acids, from 72 586.1 (Malen’kii Printz) to 2938.8 (Ukrainka) for polyols, from 2865.9 (Orlovskii Val’s) to 357.5 (Volshebnitsa) for free fatty acids, from 706 650.7 (Malen’kii Printz) to 111 403.2 (Aleander) for hexoses, and from 321 665.0 (Tisel) to 16 001.9 (Aleander) for oligosaccharides. A narrower range was recorded for free amino acids: from 439.4 (Dobriyi Dzhinn) to 95.2 (Slavyanka), from 5841.0 (Malen’kii Printz) to 1929.41 (Orlovskii Val’s) for pentoses, from 7087.0 (Malen’kii Printz) to 1432.1 (Orlovskii Val’s) for PCCs, and from 4082.6 (Aleander) to 1167.8 (Orlovskii Val’s) for sugar derivates (Fig. 1).

The metabolomic profiles of R. nigrum and L. caerulea differed in terms of representation of different groups of compounds. Mono-, oligosaccharides, free fatty acids, and lactone forms of organic acids dominated in blackcurrant MPs, while organic acids, polyols, PCCs, and free amino acids dominated in honeysuckle (see Fig. 1, Suppl. Material 1). Sugar derivatives were present in almost equal amounts in the MPs of these berry crops (see Fig. 1). The content of organic acids was higher in honeysuckle MPs due to significant amounts of malic and quinic acids (see Fig. 1, Suppl. Material 1)

Malic and glucuronic acids dominated in blackcurrant fruits (17 501.9 and 4271.6, respectively), while glucono-1,4- lactone (802.6) dominated among lactones, dulcitol and myoinositol (28 551.3 and 1513.2) among polyols, oleic and vaccenic acids (213.3 and 211.2) among free fatty acids, fructose, glucose, galactose, sorbose (192 582.1, 151 908.7, 20 264.5, 2847.6) among monosaccharides, D-6-deoxy mannopyranoside- α-L galactofuranose (1526.9) among sugar derivatives, and shikimic acid and quercetin (451.4 and 278.6 ppm) among PCCs. The fruits of honeysuckle showed the dominance of malic and quinic acids (19 124.6 and 12 936.3, respectively), threono-1,4-lactone (265.9), dulcitol and mannitol (27 526.1 and 12 983.1), palmitic acid (111.0), fructose, glucose, galactose, arabinose (122 033.3, 110 907.9, 18 046.9 and 3165.4), 2-O-glycerol-α-galactopyranoside, quinic acid and antirrinoside (2392.3, 12 936.3 and 1209.0 ppm, respectively). In currant and honeysuckle MPs, hydroxyproline prevailed in the group of free amino acids (203.6 and 254.6 ppm, respectively), and sucrose dominated in the group of disaccharides (139 416.6 and 39 660.7) (see Suppl. Material 1).

An average degree of variability (20–33 %) in blackcurrant MPs was established for succinic and threonic acids, for ribose and gallocatechin, while in honeysuckle MPs it was established for lactic, phosphoric, succinic, erythronic, threonic, glyceric, aconitic acids, for dulcitol, erythritol, myo-inositol, ribose, fructose, sorbose, galactose, mannose, glucose, glycerol-3-phosphate, arbutin, and 1,2,3,6-tetrahydropurine- 2,6-dione.

A high degree of variability (33–60 %) in blackcurrant MPs was determined for fumaric, malic, erythronic, ribonic, quinic, 4-hydroxycinnamic, ascorbic, gallic, palmitic acids, for erythrono- 1,4-, threono-1,4-, xylonolactones, leucine, oxyproline, myo-inositol, galactinol, glyceroaldehyde, arabinose, fructose, galactose, mannose, glucose, melibiose, sucrose, stachyose, glycerol-3-phosphate, α-methyl glucofuranoside, methylrutinose, 6-deoxy-mannopyranoside-α-galactofuranose, catechin, epigallocatechin, and quercetin. In honeysuckle, a high degree of variability was found for fumaric, maleic, ribonic, quinic, glucuronic, 2-keto-gluconic, caffeic, oxalic, benzoic, palmitic, stearic acids, for chlorogenic acid and its isomers, glucono-1,4-lactone, arabinitol, mannitol, quercetin, glyceraldehyde, arabinose, xylose, sucrose, rutinose, turanose, and α-methyl glucofuranoside.

A very high degree of variability (above 60 %) in blackcurrant MPs was noted for lactic, nicotinic, citraconic, glyceric, aconitic, glucuronic, 2-keto-gluconic, caffeic, galactopyruronic, palmitic, vaccenic acids, for glycerol, isomers of inositol, sorbose, 2-O-glycerol-α-galactopyranoside, α-tocopherol, scopolin, while in honeysuckle MPs it was noted for pyruvic, nicotinic, citraconic, malic, protocatechuic, α-ketoglutaric, pipecolic, linoleic, oleic acids, for threono-1,4-lactone, glucono- 6-phosphate, galactinol, raffinose, maltose, α-methyl glucofuranoside, 2-O-glycerol-α-galactopyranoside, catechin, and antirrhinoside

The main part of the blackcurrant MP components had a high degree of variability, while the honeysuckle MP components split into almost equal groups with a slight margin in favor of those with a coefficient of variation (CV) above 33 % (Suppl. Material 2).

The metabolomic profiles of black currant and honeysuckle differed from each other in a number of parameters. The MPs of blackcurrant demonstrated significantly higher ( p = 0.05) values of organic acids (pyruvic, phosphoric, nicotinic, fumaric, threonic, 4-hydroxybenzoic, maleic, arabic, ribonic, shikimic, gluconic, 4-hydroxycinnamic, ascorbic, and gallic acids), of lactone forms of arabic and xylonic acids, erythrono- 1,4-lactone, threono-1,4-lactone, 1,4-3-ols (gallocatechin, epigallocatechin), flavonols (quercetin), and oxycoumarins (scopoline). In the honeysuckle MPs, significantly higher values were observed for succinic, erythronic, glyceric, aconitic, oxalic, protocatechuic, quinic, benzoic, α-ketoglutaric, chlorogenic stearic, and pipecolic acids, for isomers of chlorogenic acid, glucono-6-phosphate, polyols (erythritol, arabinitol, mannitol, myo-inositol), monosaccharides (glycerol- 3 phosphate, arabinose, mannose), oligosaccharides (rutinose, maltose, and turanose), sugar derivatives (α-methyl pentafuranoside and 2-O-glycerol-α-galactopyranoside), flavonoids (catechin and kaempferol), glycosides (arbutin, antirrinoside, ammonium base of choline), and purine derivative 1,2,3,6-tetrahydropurine-2,6-dione. A lower degree of reliability (0.1 > p > 0.05) was demonstrated by the differences between MPs of R. nigrum and L. caerulea in terms of lactic, citraconic, galactopyranuronic acids, glyceraldehyde, sorbose, glucose, and α-tocopherol (see Suppl. Material 2).

Quantitative and qualitative differences in the MPs reflect the peculiarities of metabolism in the fruits of R. nigrum and L. caerulea. The process of accumulation of ascorbic acid, glucuronic acids, monosaccharides, especially of pentoses, fructose, mannose, galactose, as well as metabolism of free fatty acids, the Krebs cycle, glycolysis and pentose phosphate cycle are more intense in blackcurrant. The conversion of lysine along with the accumulation of pipecolic acid, the glyoxylate pathway, the exchange of phosphoric acid (phosphotransferase system) and purine bases, the synthesis of secondary metabolites (phenylpropanoids, flavonoids: flavones and flavonols) are more intense in honeysuckle. The latter is confirmed by an increase in the fraction of secondary metabolites in honeysuckle MPs up to 4.1 % compared to that in currant MPs (less than 0.5 %).

The sugars to organic acids ratio in blackcurrant and honeysuckle fruits was 15 and 7, respectively, i. e., the sugar-acid index of R. nigrum is optimal for food consumption. Honeysuckle is distinguished by high values of bioactive compounds, which makes the crop attractive as a raw material for the production of BAS, including the use of microorganism cultures.

Blackcurrant fruits contain more bioactive lactone forms of acids, mono- and oligosaccharides, which affect the taste quality of berries. The group of PCCs in blackcurrant has a better representation of 4-hydroxybenzoic, gallic, shikimic, and hydroxycinamic acids, of epigallocatechin, quercetin, α-tocopherol, scopolin, while in blue honeysuckle these are benzoic, protocatechuic, quinic, and chlorogenic acids, isomers of chlorogenic acid, catechin, arbutin, antirrinoside and kaempferol. Phenol-containing substances are anti-stress factors that constitute a part of the antioxidant defense system of plants. Most of the identified osmoprotective polyols are characteristic of honeysuckle MPs, while oligosaccharides with similar properties are typical of blackcurrant MPs. Free fatty acids can also be an evidence of protective mechanisms, since they indirectly reflect the activity of lipid synthesis, which are part of the membrane complex. The honeysuckle MPs were found to contain such an anti-stress factor as a non-protein pipecoline amino acid. A relatively low content of organic acids and the high content of sugars in the MPs of blackcurrant fruits, which influences the palatable attractiveness of fruits, may be associated with the breeding process aimed at improving the nutritional qualities of the created varieties.

**Fig. 2.**
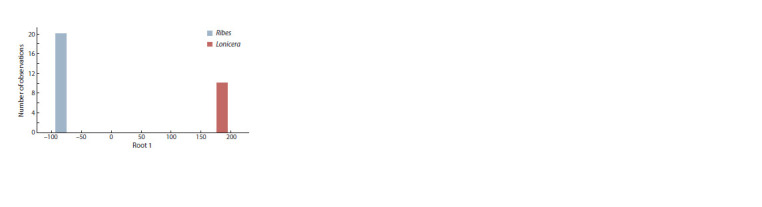
Histogram of R. nigrum and L. caerulea fruit samples distribution according to the magnitude of the eigenvalues of the canonical variable.

The canonical discriminant analysis of the obtained results confirms the difference between R. nigrum and L. caerulea species at the MP level. The most ʻinformatively valuableʼ traits that confirmed the individuality of MPs of R. nigrum and L. caerulea with an accuracy up to 98 %, were indicators of phosphoric, nicotinic, succinic, 4-hydroxybenzoic, glyceric, arabic, ribonic, protocatechuic, ascorbic, gallic, caffeic, oxalic, benzoic acids and glyceraldehyde. These compounds are involved in the main reactions of primary and secondary metabolism in plant tissues, i. e. the Krebs cycle, redox reactions, glyoxylate cycle, glycolysis, and shikimate pathway of PCC biosynthesis (Fig. 2, Suppl. Material 3). The histogram of the canonical variable eigenvalues distribution shows that the value approaches –100 for R. nigrum accessions and 200 for L. caerulea (see Fig. 2, Suppl. Material 3).

The cluster analysis using the Ward method, taking into account all the identified compounds, showed that the honeysuckle accessions were divided into two clusters (Suppl. Material 4, a). The first one consisted of two subclusters, one of which included accessions with a predominance of polyols and oligosaccharides in the MPs (‘Leningradsky Velikan’ and ‘838-12’), while the other included those with a predominance of free amino acids (‘Avacha’, ‘Start’, ‘Suvenir’, ‘Malvina’, and ‘Morena’). The next cluster was formed by honeysuckle varieties with high levels of organic acids, pentoses, hexoses, glycosides, free fatty acids, and PCCs (‘Bazhovskaya’, ‘S 322-4’, and ‘Solovey’). Black currant varieties with high levels of organic acids and sugar derivatives (‘Aleander’, ‘Orlovskaya Serenada’, and ‘Charovnitsa’) were included in the same cluster with blue honeysuckle accessions. The other blackcurrant varieties formed their own cluster, divided into two subclusters. The first included the bulk of the accessions with a high content of free amino acids, free fatty acids, pentoses, hexoses, polyols, and PCCs (‘Mila’, ‘Volshebnitsa’, ‘Malen’kii Printz’, ‘Azhurnaya’, ‘Zhuravushka’, ‘Orlovskii Val’s’, ‘Muravushka’, ‘Krasa L’vova’, ‘Cherechneva’, ‘Biryusinka’, and ‘Slavyanka’), while the second one united accessions with high values of free amino acids and oligosaccharides (‘Dobriyi Dzhinn’, ‘Margo’, ‘Syuita Kievskaya’, ‘Pamyati Potapenko’, ‘Tisel’, and ‘Ukrainka’).

A more precise separation of R. nigrum and L. caerulea accessions was achieved by taking into account the results of the PCC group (Suppl. Material 4, b). Blackcurrant and blue honeysuckle accessions formed two separate clusters, each of which, in turn, was divided into two subclusters. The first subcluster consisted of honeysuckle accessions with high levels of flavones and phenylpropanoids (‘Leningradsky Velikan’, ‘Solovey’, and ‘838-12’); while the second one included those with high levels of glycosides, flavan- 3-ols, flavanones, and benzoic acid derivatives (‘Avacha’, ‘Start’, ‘Suvenir’, ‘Malvina’, ‘Morena’, ‘Bazhovskaya’, and ‘S 322-4’). A separate subcluster was formed by blackcurrant varieties with high values of all the identified PCCs (‘Tisel’, ‘Charovnitsa’, ‘Margo’, ‘Pamyati Potapenko’, ‘Cherechneva’, and ‘Malen’kii Printz’).

The study has identified blue honeysuckle varieties with a high content of certain groups of compounds: ‘Bazhovskaya’ (PCCs), ‘S 322-4’ (organic acids, free fatty acids and monosaccharides), ‘Leningradsky Velikan’ (oligosaccharides), ‘Malvina’ (free amino acids), and blackcurrant varieties: ‘Malen’kii Printz’ (monosaccharides, PCCs, and polyols), ‘Dobriyi Dzhinn’ (free amino acids), ‘Tisel’ (oligosaccharides), ‘Orlovskii Val’s’ (free fatty acids), and ‘Aleander’ (organic acids and sugar derivates).

## Discussion

We compared our data with the results of other studies. The current experiment confirmed that the total content of phenolic compounds in honeysuckle fruits is higher and their qualitative composition is different from other crops, which was previously established by VIR researchers (Streltsina et al., 2005–2007). It was also noted in the mentioned works that the high content of phenolic compounds in honeysuckle is due to its recent inclusion in the breeding process and the great similarity of the created varieties of this crop with its wild relatives. This is also confirmed by our data.

In contrast to the results obtained by Sochor et al. (2014) and Gołba et al. (2020), according to which hydroxycinnamic acids and flavonols dominate among the PCCs of L. caerulea, quinic acid was best represented in this group of compounds in our study, and the content of chlorogenic acid and its derivatives was significantly lower. The data on the iridoid glycoside (antirrhinoside) identified in the honeysuckle fruits studied in the present work are consistent with the results of Senica et al. (2018) and Gołba et al. (2020), but contradict those of Sochor et al. (2014). We identified only hydroxyproline and leucine in the group of free amino acids, which disagrees with the study by Sochor et al. (2014). The composition of organic acids and sugars in the honeysuckle fruit samples studied by us corresponds to the data from the works by Rop et al. (2011), Sochor et al. (2014), Senica et al. (2018), Gołba et al. (2020), and Juríková et al. (2020).

The publications of VIR researchers (Streltsina et al., 2005; Tikhonova, Streltsina, 2009, 2012; Streltsina, Tikhonova, 2010; Tikhonova et al., 2015) report on such economically important features of blackcurrant as the optimal sugar-acid index and high pectin values, which is confirmed by our results concerning the ratio of sugars and acids in the fruits of R. nigrum and L. caerulea, and the presence of uronic acids in the MPs of R. nigrum. According to Lee et al. (2015), and Tian et al. (2019), fructose, galactose, and glucose predominate among monosaccharides at the technical ripeness stage. Similar results were obtained in our work. According to H.J. Lee and colleagues, malonic acid dominated in the group of acids, sorbitol in the group of polyols, and quercetin and kaempferol in that of phenolic substances (Lee et al., 2015). However, this is inconsistent with our data. The paper by Tian et al. (2019) names citric and malic acids as the main organic acids in blackcurrant fruits, anthocyanins and flavanols as the main phenolic compounds, and hydroxycinnamic acids as the main phenolic acids. It was established by P.H. Mattila and colleagues that, in addition to anthocyanins, the dominant phenolic compounds in black currant are such flavonols as mirecetin and quercetin (Mattila et al., 2016). Concerning the samples studied in the present research, malic and glucuronic acids predominated in the group of organic acids, hydroxycinnamic acids and their derivatives (chlorogenic acids) in the group of phenolic acids, and shikimic acid and flavonol quercetin dominated among the PCCs. A comparative analysis of the data obtained by us with the results of other researchers revealed a number of discrepancies associated with differences in conditions for the material regeneration and methodological approaches chosen for the study. In the papers mentioned above, the authors underline the dependence of the biochemical composition of fruits on growing conditions (region), which confirms the relevance of our work (Rop et al., 2011; Sochor et al., 2014; Lee et al., 2015; Mattila et al., 2016; Senica et al., 2018; Tian et al., 2019; Gołba et al., 2020; Juríková et al., 2020).

The study of R. nigrum and L. caerulea accessions from the VIR collection within the framework of the joint international BacHBerry project confirmed the use of honeysuckle as a donor of genes controlling the biosynthesis of secondary metabolites to be promising for the creation of microbiological producers of natural bioactive substances (Thole et al., 2019).

## Conclusion

The performed work made it possible to define features of metabolomic profiles of R. nigrum and L. caerulea berry crops grown in conditions of the Leningrad Province, to identify varieties with economically important traits, suitable for expanding the range of functional, therapeutic and prophylactic food products (‘S 322-4’, ‘Leningradsky Velikan’, ‘Malvina’, ‘Malen’kii Printz’, ‘Dobriyi Dzhinn’, ‘Tisel’, and ‘Orlovskii Val’s’), for producing bioactive supplements and medicines based on natural bioactive substances (‘Bazhovskaya’, ‘Aleander’), and for breeding aimed at creating varieties that combine nutritional advantages with resistance to environmental stress factors (‘Bazhovskaya’, ‘S 322-4’, ‘Leningradsky Velikan’, ‘Malen’kii Printz’, ‘Tisel’, and ‘Aleander’).

## Conflict of interest

The authors declare no conflict of interest.
